# The Productivity Dynamics of China's Environmentally Friendly Production Technologies in terms of Wastewater Treatment Techniques

**DOI:** 10.1155/2018/6878741

**Published:** 2018-03-29

**Authors:** Fuxia Yang, Mian Yang, Jiangchuan Xu

**Affiliations:** ^1^College of Economics & Management, Huazhong Agricultural University, Wuhan 430070, China; ^2^Economics and Management School, Wuhan University, Wuhan 430072, China; ^3^Center of Population, Resource & Environmental Economics Research, Wuhan University, Wuhan 430072, China

## Abstract

Low economic profit usually reduces the incentive of producers to operate their wastewater treatment technologies effectively. It is necessary to investigate the performance of environmentally friendly production technologies that reduce wastewater discharges and generate economic outputs simultaneously (EPTWs) in China over the past decade. In this paper, we apply the Malmquist-Luenberger productivity index widely used in the field of economics to evaluate the productivity change of EPTWs for 30 administrative provinces in China during 2003–2015. The pathways of the productivity change are further identified by decomposing the productivity index into two components: technological change and technical efficiency change. The results show that China's environmental productivity index associated with wastewater reduction had undergone a downward trend, and evident spatial disparities are observed among the 30 provincial regions. Moreover, the changes of China's environmental productivity over the whole studied period can mainly be attributed to technological progress, while the technical efficiency component has contributed little, although its annual contributing rate is in an increasing trend.

## 1. Introduction

Over the past three decades, China has witnessed a dramatic economic growth with the annual average growth rate at nearly 10%. At the same time, serious pollution issues emerged in large numbers resulting in obvious environmental degradations. As one of the most important pollutants, wastewater discharge is a very serious environmental problem resulting from the rapid urbanization, the numerous usages of fertilizer, and industrial activities and residents' living consumption [1, 2]. In order to improve the worsening water environment quality, the Chinese government has enforced a series of ambitious plans to decrease pollutant discharges and promote wastewater purification since 2000. For instance, the total chemical oxygen demand (COD) in China was required to be reduced by 10% during 2006 to 2010 (the 11th five-year plan, FYP) by the central government.

**Figure 1:**
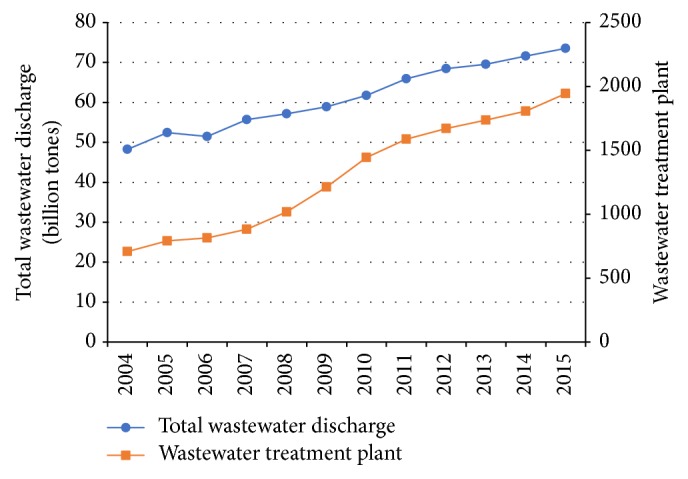
Total wastewater discharge and municipal wastewater treatment plants in China.* Data Source*:* China Environmental Statistical Yearbooks*, 2005–2016.

In this context, various wastewater treatment technologies (WTTs) and cleaner production techniques have been developed and adopted widely [3, 4]. It is reported that the number of municipal wastewater treatment plants have increased from 708 to 1944 over the period 2004–2015 (Figure 1), and the designed treatment capacity per day has been also improved by nearly two times. Nevertheless, with the continuous decline of total COD, the water environmental quality in this developing country has not been fundamentally improved. The total wastewater discharge has even observed a continuing increase with the average annual growth rate at about 4%, from 48.2 billion tons in 2004 to 73.5 billion tons in 2015 (Figure 1), and the total economic losses from water pollution in China are estimated to be around 240 billion yuan per year [5].

Numerous studies focus on the underlying reasons why the total effluent discharge kept increasing in the context of stringent wastewater management regulation. A popular point of view is that WTTs in China have not been operated efficiently [6, 7]. First, the installations of wastewater treatment facilities and pipeline networks require huge upfront capital investment [8, 9], which reduces the incentives of producers to invest in wastewater reclaim and reuse programs [4]. Second, due to the very high operating expenditure including labor input and electricity consumption during the treatment processes (accounting for more than 50% of the total economic costs) [10], the sewage treatment facilities that have been put into operation are usually running with low efficiency; some of them are even left unused. Besides, the lack of integrated water resources management framework, incoherent water quality requirements, and weak public awareness also impede the adoption of WTTs [4]. By contrast, the economic returns of these technology adoptions are limited. In recent years, various advanced wastewater treatment techniques have been developed, by which the capital and operating costs have been remarkably reduced [3, 4, 11]. Since the benefits related to the adoption of WTTs accrue the unequal distribution among the stakeholders [12], the revenue gained from the implementation of WTTs is far from their total costs [13]. It can be inferred from previous studies that low economic profit is the crucial factor of inefficient use of current advanced WTTs. In practice, producers aiming to maximize their profits prefer environmentally friendly production technologies that generate economic revenues and reduce sewage discharges simultaneously (EPTW) in the face of stringent environmental regulation. Therefore, it is of great importance to evaluate the performance of EPTWs.

In this paper, we attempt to evaluate the level of EPTWs among 30 administrative provinces in China by constructing an environmentally sensitive productivity index. This productivity index captures the intertemporal change of productivity for EPTWs by examining the actual effect of economic returns and sewage reduction. The rest of this paper is organized as follows: Section 2 presents a literature review on environmental productivity analysis; Section 3 elaborates the methodology used in this study; Section 4 examines the intertemporal changes, spatial differences, and the main drivers of EPTWs' productivity for 30 administrative provinces in China; Section 5 concludes the paper and puts forward some useful policy recommendations.

## 2. Literature Reviews

The main purpose of productivity analysis is to evaluate the performance of one production technology in terms of economic gains. Traditional total factor productivity index (namely, Solow residual) is defined as outputs change not explained by input variations, which has gained popularity in the field of economics. However, the initial analysis framework ignores undesirable outputs in the production process, which fails to provide a full picture of sustainable economic development. With the continued deterioration in global environmental condition, more attention has been paid to green economic growth. To this end, Chung et al. [14] propose a Malmquist-Luenberger productivity index (ML index) based on a directional distance function. The ML index measures environmentally sensitive productivity change through incorporating undesirable byproducts of economic outputs.

Ever since this seminal work, the ML index has been widely used to analyze the change of productivity for a wide range of decision-making units (DMUs) with bad outputs. Färe et al. [15] compute the ML index of US state manufacturing sectors from 1974 to 1986 considering both marketed output and air pollution emissions. Hailu and Veeman [16] investigate the environmentally sensitive productivity change of the pulp and paper industry in Canada by employing a parametric input distance function. Yörük and Zaim [17] evaluate ML productivity indicator taking carbon dioxide, nitrogen oxide, and organic water into account. Mahlberg et al. [18] estimate environmental productivity change including greenhouse gas emissions as an undesirable output. Ananda and Hampf [19] measure environmentally sensitive productivity growth of the urban water sector.

With the increase of environmental degradation, the evaluation on China's environmental productivity has also attracted more and more attention from the scholars. The majority of available literature investigates environmentally friendly production technologies related to sulfur dioxide (SO_2_), chemical oxygen demand (COD) and carbon dioxide (CO_2_). For instance, some researches model CO_2_ emissions as the undesirable output to analyze environmental productivity change of Chinese manufacturing industries [20–22], industrial sectors [23, 24], transportation industry [10, 25], and iron and steel industry [26]. Yang et al. [27] and F. Yang and M. Yang [28] incorporate SO_2_ emissions as the bad output when computing environmental productivity index of 30 provincial regions in China. Being aware of the severity of water pollution issue, several studies try to take sewage discharge or the main pollutants in wastewater into account in their environmental productivity assessments. He et al. [29] measure the bad output of waste gas, wastewater (including biochemical oxygen demand and total suspended solids) and solid wastes to compute the ML productivity index of iron and steel industry. Tao et al. [30] and Xie et al. [31] assess green productivity growth by employing global ML productivity index, where wastewater discharge, SO_2_ emissions and soot serve as undesirable outputs.

Furthermore, deteriorating water quality turns some scholars' attention to assess water use efficiency considering undesirable outputs. For example, Wang et al. [32] investigate water use efficiency of China's regional industrial systems, taking two main pollutants in wastewater (i.e., COD and ammonia nitrogen) into account. Deng et al. [33] estimate water use efficiency of 31 provinces in China during 2004–2013 using slack based measure-data envelopment analysis (SBM-DEA) model, which takes consideration of sewage. Zhao et al. [34] incorporate COD in industrial wastewater and ammonia nitrogen output of urban sewage when water resource utilization efficiency for 31 provincial administrative regions in China is measured. These models make it possible to compare the environmental efficiency of water use for cross-sectional DMUs at a certain time. However, they do not describe the intertemporal change of water efficiency scores for one producer over time. Second, these models can say very little about the sources of changes in the efficiency scores.

This paper extends prior literature on the water utilization efficiency by attempting to address the above-mentioned two limitations. In the following analysis, we construct an environmental productivity index to evaluate the dynamic change of technical efficiency considering wastewater discharge reduction. Moreover, the productivity index allows us to identify the driver of the productivity change of EPTWs for one production unit by decomposing it into technical change (the shift of the water-friendly production frontier) and efficiency change (the move toward the water-friendly frontier).

## 3. Methodology

### 3.1. Environmental Directional Distance Function

To construct an environmentally sensitive productivity index in terms of wastewater reduction and economic gains (EPI_WE_), it is necessary to define an environmental directional distance function first. Then consider an environmental production technology where one production unit uses a vector of input **x** = (*x*_1_, *x*_2_,…, *x*_*n*_) ∈ *R*_*n*_^+^ to yield economic outputs **y** = (*y*_1_, *y*_2_,…, *y*_*m*_) ∈ *R*_*m*_^+^ and discharge wastewater **u** ∈ *R*^+^. The production possibility set (PPS) for this production technology, **P**(**x**), can be expressed as **P**(**x**)* = *{(**y**, **u**) : **x**  can  produce  (**y**, **u**)}, which satisfies a set of axioms discussed in [35]. In addition, inputs and economic outputs are strongly disposable, and discharged wastewater is weak disposable.

To distinguish a specific production behavior such as input saving, economic growth, three kinds of the distance function including input-oriented, output-oriented, and directional one are developed to alternatively describe the PPS. The directional distance function has been widely used since the other two can be considered as its special case. Furthermore, the attractive merit is that it can expand desirable outputs and contract inputs/undesirable outputs simultaneously. Here, to recognize production activities that are friendly to wastewater reduction, the environmental directional distance function is defined as follows:

(1) D→x,y,u;g=sup⁡β:x,y+β·gy,u−β·gu∈P,

where **g** = (*g*_*y*_, −*g*_*u*_) is the vector of directions which economic outputs and wastewater are scaled. To measure technical efficiency defined in the Farrell proportional distance function, we define **g** = (*y*, −*u*), by which wastewater and desirable outputs are proportionately adjusted. Obviously, this distance function determines the benchmark of EPTWs, where producers yield the maximum of economic outputs *y* + *β* · *g*_*y*_ but discharge the least of wastewater *u* − *β* · *g*_*u*_ with inputs held fixed. Accordingly, *β* is the proportions of maximum feasible increase in economic outputs and decrease in discharged wastewater towards the production frontier of EPTW. They take values larger than or equal to zero. The larger this value takes, the wider the gap between its current technology and the best practice is. Since the directional vector has been chosen at the observed outputs, the value of the distance function *β* is independent of measurement units for output variables [28, 36].

### 3.2. Environmental Productivity Index and Its Decomposition

According to Chung et al. [14], the above-defined directional distance function can be used to construct an environmentally sensitive productivity index (namely, EPI_WE_). To avoid the impact of arbitrarily employing a reference technology on the final result, EPI_WE_^*t*,*t*+1^ is defined as follows:

(2) EPIWEt,t+1=1+D→txt,yt,ut1+D→txt+1,yt+1,ut+1·1+D→t+1xt,yt,ut1+D→t+1xt+1,yt+1,ut+11/2,

where ${\vec{D}}^{t}(x^{t},y^{t},u^{t})$ and ${\vec{D}}^{t+1}(x^{t+1},y^{t+1},u^{t+1})$ are contemporaneous environmental directional distance functions capturing the distance of the observed input-output combination of one producer from the current best practice. ${\vec{D}}^{t}(x^{t+1},y^{t+1},u^{t+1})$ and ${\vec{D}}^{t+1}(x^{t},y^{t},u^{t})$ refer to cross-period distance functions, which compare the observed data at period *t* + 1 (or *t*) to the frontier at period *t* (or *t* + 1). Here EPI_WE_^*t*,*t*+1^ measures the intertemporal changes of the productivity level related to the EPTWs. EPI_WE_^*t*,*t*+1^ > 1 shows the productivity improvement for one production unit in period *t* + 1 compared to period *t*. On the contrary, EPI_WE_^*t*,*t*+1^ < 1 indicates productivity decline; that is, this producer uses some technologies not friendly to wastewater reduction and economic growth in period *t* + 1. It is worthwhile to note that there may exist productivity decline when the speed of one production unit undertaking wastewater reduction activities is slower than that of the frontier technology. EPI_WE_^*t*,*t*+1^ = 1 indicates a relative stagnation in the EPTW's productivity level.

In general, one production unit can enhance its productivity of EPTWs by several measures, such as upgrading treatment technologies and optimizing the current technology process. To identify the drivers of the change in environmentally sensitive productivity, the EPI_WE_ index can further be decomposed into two components: technological change (EPITC_WE_) represented as the shift in the EPTWs' frontier and technical efficiency change (EPIEC_WE_) as the move towards the best practice:

(3) $$\begin{array}{c} {EPI}_{WE}^{t,t+1}=EPITC_{WE}^{t,t+1}\cdot EPIEC_{WE}^{t,t+1}, \end{array}$$

where

(4) EPITCWEt,t+1=1+D→t+1xt,yt,ut1+D→txt,yt,ut·1+D→t+1xt+1,yt+1,ut+11+D→txt+1,yt+1,ut+11/2,EPIECWEt,t+1=1+D→txt,yt,ut1+D→t+1xt+1,yt+1,ut+1.

Here EPITC_WE_^*t*,*t*+1^ measures the geometric mean of the shift in the frontier of the EPTW in each two-year period. EPITC_WE_^*t*,*t*+1^ > 1 indicates the technological progress of EPTWs, resulting from the innovation activities that attain economic growth and wastewater reduction simultaneously. EPITC_WE_^*t*,*t*+1^ < 1 and EPITC_WE_^*t*,*t*+1^ = 1 reflect a regress and relative stagnation of the EPTW frontier, respectively. EPIEC_WE_^*t*,*t*+1^ is the change of distances between the used technologies and the current EPTW frontier throughout two periods, which captures the speed by which producer moves towards the current best practice, namely, catch-up or fall-behind effect. EPIEC_WE_^*t*,*t*+1^ > 1 implies technical efficiency improvement of EPTWs, as a result of the enhancements of labors' operation skills and managerial and institutional environment with respect to the EPTW; EPIEC_WE_^*t*,*t*+1^ < 1 shows that technical efficiency declines due to deteriorated operating environment.

### 3.3. Calculation of EPI_WE_ and Its Two Components

To avoid the misspecification of the functional form often confounded by econometrics methods, this paper employs nonparametric data envelopment analysis (DEA) technique to compute the values of EPI_WE_^*t*,*t*+1^, EPITC_WE_^*t*,*t*+1^, and EPIEC_WE_^*t*,*t*+1^. Given that the technologies developed in previous periods are still feasible in the following years, sequential DEA technique is used to construct the best practice with its main merit of eliminating the possibility of registering any technical regress by definition [28, 37]. Then the benchmark at period *t* should be ${\bar{T}}^{t}=T^{1}\cup T^{2}\cup \cdots \cup T^{t}$, where *T*^*t*^ derived from the observed data of a set of *N* entities at time *t* can be written as follows:

(5) Ttxt,yt,ut=xt,yt,ut:xt  can  produce  yt,ut=xt,yt,ut:∑i=1Nzitxkit≤xkit,  ∑i=1Nzitymit≥ymit,  ∑i=1Nzituit=uit,  zit≥0,  i=1,…,N,

where *z*_*i*_^*t*^ is the weight assigned to corresponding observation. The inequality constraints on the inputs and economic outputs as well as the equality constraint on discharged wastewater reflect their strong and weak disposability, respectively.

For the *i*th producer at time *t*, the contemporaneous distance function ${\overset{\to }{D}}^{t}(x^{t},y^{t},u^{t};g^{t})$ can be calculated by solving the following linear programming:

(6) D⟶txt,yt,ut;gt=max βit,ts.t. ∑s=1 t∑i=1Nzisxhis≤xkit,k=1,…,K ∑s=1 t∑i=1Nzisyis≥ymit+βit,tymit,m=1,…,M ∑s=1 t∑i=1Nzisuis=uit−βit,tuit, zis,βit,t≥0,∀i,k,m;  i=1,…,N.

The intertemporal distance function ${\overset{\to }{D}}^{t}(x^{t+1},y^{t+1},u^{t+1};g^{t+1})$ for the *i*th entity can be computed from the following linear programming:

(7) D⟶txt+1,yt+1,ut+1;gt+1=max βit,t+1s.t. ∑s=1 t∑i=1Nzisxhis≤xkit+1,k=1,…,K ∑s=1 t∑i=1Nzisyis≥ymit+1+βit,t+1ymit+1,m=1,…,M ∑s=1 t∑i=1Nzisuis=uit+1−βit,t+1uit+1, zis,βit,t+1≥0,∀i,k,m;  i=1,…,N.

Likewise, ${\overset{\to }{D}}^{t}(x^{t+1},y^{t+1},u^{t+1};g^{t+1})$ and ${\overset{\to }{D}}^{t+1}(x^{t},y^{t},u^{t};g^{t})$ can also be calculated by solving a similar linear programming.

## 4. Results and Discussions

### 4.1. Data Descriptions

The study sample of this paper contains 30 inland administrative provinces (provinces, autonomous regions, and municipalities) in China during 2002–2015. Due to the absence of basic data on wastewater discharge, the Tibet autonomous region is not included in current study.

As shown in (6) and (7), there are three kinds of factor inputs (including capital, labor, and water) and two kinds of outputs (one desirable output and one undesirable output). Here the amounts of capital input (*K*) and labor input (*L*) are represented by capital stock and employed persons, respectively, while the water resource input (*W*) is measured by regional total water consumption. Besides, real GDP and total wastewater discharges (WD) serve as the proxies of desirable output and undesirable output, respectively.

First of all, the amount of province-level capital stock has not been reported in any China's official statistics. Following previous studied [38], we calculate it using perpetual inventory method. The data on employed persons is collected from annual* China Statistical Yearbooks* and* China Regional Economics Statistical Yearbook* (CRESY, 2007). The basic data on provincial total water consumption during 2002-2003 and 2004–2015 can, respectively, be obtained from* China Environmental Statistical Yearbooks* (CESYs, 2003-2004) and* China Statistical Yearbooks* (CSYs, 2005–2016). The real GDP is acquired from* China Statistical Yearbooks* (CSYs, 2003–2016) by deflating its nominal value with the GDP deflator, and the wastewater discharges are also collected from* China Statistical Yearbooks *(CSYs, 2003–2016). Furthermore, both real GDP and capital stock are measured with 1995 price levels.

Table 1 reports the descriptive statistics of the five variables over the whole study period. In general, the mean values for all the variables are much larger than the median values, indicating that most regions are observed near the left tail of the distribution. Moreover, the standard deviation is less than the mean value, showing that there are no outliers for all series.

### 4.2. Spatial-Temporal Variations of China's EPI_WE_

The results of environmental productivity index concerning both economic growth and wastewater reduction for each administrative province as well as the national average during 2003–2015 are reported in the Appendix.

#### 4.2.1. Intertemporal Change of China's Environmental Productivity Index

**Figure 2:**
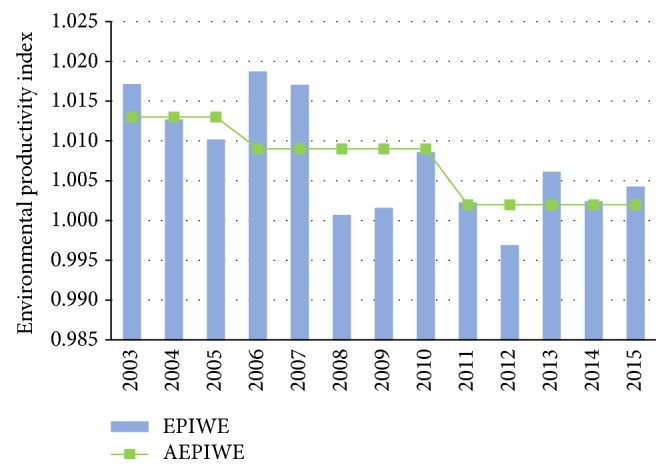
Intertemporal change of China's EPI_WE_.* Note*. AEPI_WE_ means the average EPI_WE_ in each period.

According to the results shown in Table 2, the dynamic feature of China's EPI_WE_ can be investigated (Figure 2). Generally speaking, the environmental productivity index of national average has experienced frequent fluctuations over the past decade, which declined gradually from 1.017 in 2003 to 1.010 in 2005 and then increased in 2006 due to the implementation of China's energy saving and emissions reduction policy. After that, the productivity index dropped sharply from 1.017 in 2007 to 1.001 in 2008 and then went up again from 1.002 in 2009 to 1.009 in 2010. With the overfulfillment of the target of ten percent COD reduction in the 11th five-year plan (FYP), the stress for emissions reduction from the central government temporarily relieved. In this context, the environmental productivity index declined greatly from 1.009 in 2010 to 1.002 in 2011 and even to 0.997 in 2012. In order to accomplish the target of COD reduction in the 12th FYP (2011–2015), the State Council issued* the Most Stringent Water Management System *in 2012, in which the strict system of limiting pollution discharges in water function area has been established. With the execution of a series of pollution control polices including administrative interventions, technical progress, and economic instruments, the water desirable productivity index went up consequently in the last three years of the 12th FYP.

Although frequent fluctuations for China's environmental productivity index over the whole study period have been identified, EPI_WE_ has been generally performing in a decline trend from a more long-term perspective (Figure 2). In particular, it was at a relatively high level (with the mean value at 1.013) in the last three years of the 10th FYP; after that the EPI_WE_ decreased in the 11th FYP (2006–2010) with the mean value at 1.009, and then it continued to decline in the 12th FYP (2011–2015) with its mean value at merely 1.002. According to the definition of EPI_WE_ in the Section 3, the descending EPI_WE_ greater than one (with the sole exception in 2012) during different periods means that although the improvement of environmental productivity in China is observed through the whole studied period, its rate slowed down over time.

#### 4.2.2. Regional Difference of China's EPI_WE_

**Figure 3:**
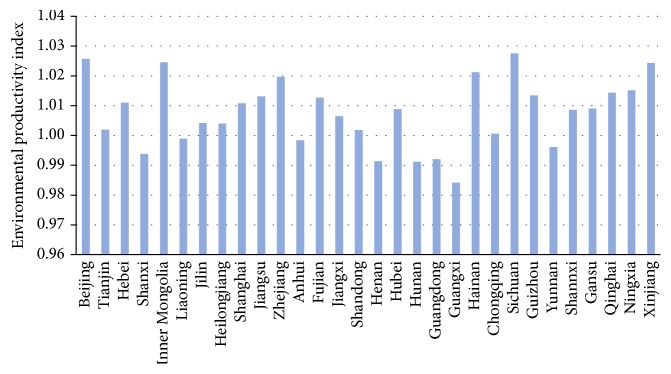
Spatial variations of China's EPI_WE_.

The spatial variations of China's environmental productivity index related to wastewater reduction during 2003–2015 are also examined (Figure 3). As we observe from Figure 3 that nearly three-fourths of the studied administrative provinces have achieved environmentally preferable productivity improvements over the whole study period. In particular, the productivity index in six provinces such as Sichuan, Beijing, Inner Mongolia, Xinjiang, Hainan, and Zhejiang has achieved significant increases with their annual mean values surpassing 1.02; seven provinces including Ningxia, Qinghai, Jiangsu, Fujian, Guizhou, Shanghai, and Hebei have also gotten moderate environmental productivity improvements with their annual mean values within 1.01–1.02; nine administrative provinces such as Hubei, Shannxi, Gansu, Jiangxi, Jilin, Heilongjiang, Tianjin, Shandong, and Chongqing saw little water desirable productivity improvement with their annual mean values within 1.00–1.01. In contrast, EPI_WE_ in the rest eight provinces (including Guangxi, Henan, Hunan, Guangdong, Shanxi, Yunnan, Anhui, and Liaoning) has undergone decrease by different extents. It can be concluded from the definition of environmental productivity index analyzed above that the actual performance of EPTWs in most of the administrative provinces had been meliorated.

### 4.3. Decompositions on China's EPI_WE_ Variations

**Figure 4:**
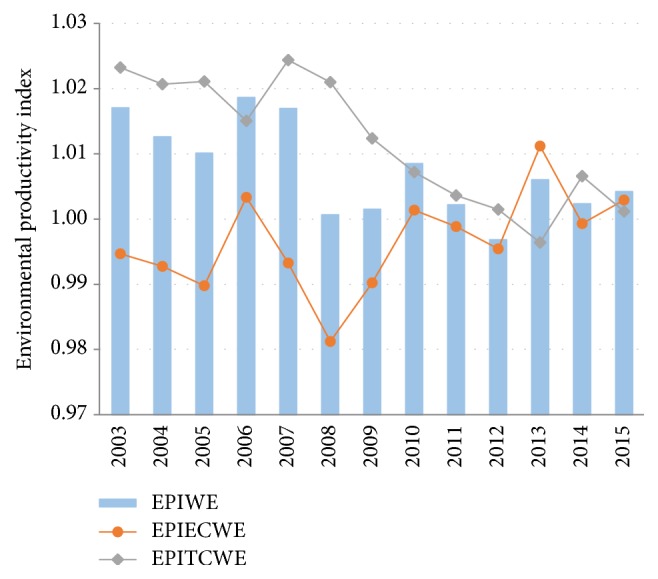
Decomposition on China's EPI_WE_.

Based on (4), the intertemporal changes of China's water preferable productivity index are decomposed and the main drivers of productivity change for all the studied regions are further identified. As shown in Figure 4, the mean values for EPITC_WE_^*t*,*t*+1^ component are higher than that of EPI_WE_^*t*,*t*+1^ in most of the years (excluding 2006, 2010, 2013, and 2015), which implies the improvements of environmental productivity with respect to wastewater reduction over the whole studied period can mainly be attributed to the shift of water desirable production technology frontier, as a result of innovation activities associated with water-saving and wastewater purification. By contrast, the mean values of the EPIEC_WE_^*t*,*t*+1^ component are persistently lower than that of EPI_WE_^*t*,*t*+1^ (with the sole exception in 2013), demonstrating that the technical efficiency change has overall impeded the improvements of China's water desirable productivity. Besides, another obvious evolution trend can be observed: the gap between the two components, that is, EPITC_WE_^*t*,*t*+1^ and EPIEC_WE_^*t*,*t*+1^, was very large before 2009; then it began to narrow since 2010, and the mean values of the technical efficiency change component were even higher than that of the technical change component in 2013 and 2015. It can be inferred from this evolution trend that the technical efficiency improvement will play a more and more important role in the enhancement of water preferable productivity in the future.

The decompositions on the intertemporal changes of cross-region environmental productivity index are also conducted. For the sake of saving space, we divide the whole study period into three substages, that is, 2003–2005 (the last 3 years in the 10th FYP), 2006–2010 (the 11th FYP), and 2011–2015 (the 12th FYP). The variation processes of the cross-region environmental productivity index decompositions are shown in Table 3. 

In the first stage (2003–2005), the EPITC_WE_ index is significantly higher than the EPIEC_WE_ index for most of the regions with the sole exception of Guangxi Zhuang Autonomous Region. In the second stage, the gap between the two components began to narrow, which is highly consistent with the variation trend of the whole country. In the third stage, the gap between EPITC_WE_ and EPIEC_WE_ further shrinks; and the EPITC_WE_ in some of the studied regions is even lower than the EPIEC_WE_, such as in Jiangxi, Chongqing, Sichuan, Ningxia, and Xinjiang. It can be well interpreted by the rising marginal abatement cost of wastewater treatment technologies. As the low-hanging fruits are picked, the potential of promoting wastewater reduction by means of adopting environmentally friendly technologies is getting less and less, and more attention should be paid to the approach of enhancing management skills and institutional renovations.

## 5. Conclusions and Policy Implications

In the face of China's deteriorated water environmental quality, the central government had formulated and carried out a series of environmental regulation policies for effluent discharge reduction during the past one and a half decades. Although the total emissions for some key indicators such as COD and ammonia nitrogen are in a decline trend, the overall water environmental quality in this developing country has not been fundamentally improved, and the total wastewater discharge has even increased by nearly 50% during the last decade. Low economic profit is considered the main barrier for firms to operate wastewater treatment technologies effectively.

In this study, we aim to evaluate the productivity change of EPTWs for 30 provincial regions in China during 2003–2015 using the ML index. To clearly identify the dominant drivers of the productivity change, this productivity index is further decomposed into two components including technological change and technical efficiency change. The results indicate that, first, the average environmental productivity index nationwide has experienced frequent fluctuations over the whole study period; when we further compare the mean values of EPI_WE_ among different substages, this key index has been generally performing in a decline trend (the mean values were 1.013 in the last three years of the 10th FYP, 1.009 in the 11th FYP, and 1.002 in the 12th FYP, resp.). Second, there exist significant spatial variations of China's environmental productivity index. In particular, the EPI_WE_ in Sichuan, Beijing, and Inner Mongolia had achieved great increases, while it had undergone decrease by different extents in eight administrative provinces such as Guangxi, Henan, and Hunan. Last but not least, the improvements of China's water desirable productivity over the whole studied period can mainly be attributed to technological innovation activities, while the technical efficiency change has contributed relatively little. However, as the low-hanging fruits have been picked, the technical efficiency component is expected to play a more and more important role in the future.

According to the conclusions drawn above, we put forward the following policy recommendations: (1) given that the total discharges of some key indicators for wastewater pollutant, such as COD and ammonia nitrogen, have been well controlled, more attention should be paid to the total sewage discharge reduction. Otherwise, the water environment quality in China can hardly be improved fundamentally without a clear and powerful environmental regulation policy. (2) Since the marginal abatement cost for wastewater discharge through engineering technology approaches (the technological change component) becomes higher and higher, more importance should be given to industrial restructuring as well as management institutional innovation (the technical efficiency component) from a long run. In this way, the abatement cost for effluent discharge can be well controlled, and the performance for wastewater treatment technologies can be enhanced. (3) The stringent wastewater environmental regulations should be persistently implemented without any loosening. In this respect, some market-based policies such as the labeling instrument can be taken into account since they encourage voluntary actions by enterprises and consumers to undertake wastewater treatment activities [39–41].

## Figures and Tables

**Table 1:** Descriptive statistics of the variables, 2002–2015.

Variables	Units	Mean	Std.D.	Min	Max	Median	Obs.
*K*	Billion Yuan	2755	2518	129	13882	1977	420
*L*	Thousand Person	24667	16325	2470	66360	20605	420
*W*	Billion Cubic meters	20	14	2	59	18	420
*Real GDP*	Billion Yuan	900	849	32	4795	643	420
*WD*	Million tce	1962	1580	111	9115	1491	420

**Table 2:** Province-level productivity index of EPTW during 2003–2015.

Regions	2003	2004	2005	2006	2007	2008	2009	2010	2011	2012	2013	2014	2015
Beijing	1.043	1.134	1.028	1.038	1.184	1.006	1.110	0.959	1.026	0.848	0.977	0.981	1.000
Tianjin	1.000	1.023	1.000	1.000	1.000	1.002	1.000	1.000	1.000	1.000	1.000	1.000	1.000
Hebei	1.028	0.995	1.046	1.041	1.031	1.006	1.003	1.008	1.001	0.985	0.999	0.997	1.003
Shanxi	1.043	1.030	0.996	0.954	1.011	0.969	0.984	0.988	1.013	0.974	0.993	0.975	0.990
Inner Mongolia	1.007	1.052	1.066	1.070	1.012	1.012	1.043	0.956	1.010	1.026	1.017	1.012	1.036
Liaoning	1.011	1.000	1.000	1.000	1.012	0.962	1.013	1.024	0.999	0.995	1.005	0.963	1.002
Jilin	1.021	1.033	0.987	0.944	1.007	0.998	1.011	1.006	1.024	1.014	1.011	0.999	1.000
Heilongjiang	1.015	1.016	1.011	1.000	1.000	1.000	1.000	1.001	0.986	0.986	1.022	1.009	1.004
Shanghai	1.029	1.031	1.017	0.999	1.037	1.000	1.000	1.013	1.000	1.008	1.000	1.005	1.000
Jiangsu	1.020	1.022	1.018	0.989	1.040	1.016	1.005	1.004	0.996	0.999	1.022	1.024	1.016
Zhejiang	1.038	1.032	1.017	1.047	1.017	1.015	1.005	1.090	0.940	1.014	1.016	1.016	1.008
Anhui	1.026	1.006	1.012	0.989	1.032	0.982	0.990	1.012	1.002	1.017	0.922	0.996	0.993
Fujian	1.013	1.030	1.026	1.038	1.018	1.000	0.989	1.013	0.983	1.007	1.016	1.015	1.019
Jiangxi	0.997	0.972	0.986	0.999	0.984	1.019	0.996	1.014	0.981	1.014	1.001	1.022	1.099
Shandong	1.010	1.009	1.001	1.000	1.004	1.000	1.000	0.999	1.000	1.000	1.000	1.001	1.001
Henan	1.027	1.023	1.002	0.964	0.976	0.950	0.981	0.996	0.998	0.986	0.991	0.996	0.998
Hubei	1.020	1.038	1.027	1.017	1.031	1.014	0.998	1.007	1.009	0.966	0.991	1.001	0.996
Hunan	1.000	1.003	1.000	0.999	1.009	0.991	0.982	0.982	0.974	0.967	0.975	1.001	1.002
Guangdong	1.033	1.000	1.016	1.000	1.012	1.000	0.981	1.011	0.983	0.998	0.918	0.965	0.980
Guangxi	1.029	0.982	1.040	0.883	1.104	0.909	0.823	0.914	1.052	0.988	1.039	1.019	1.010
Hainan	1.007	0.956	1.010	1.042	1.045	1.021	1.021	1.051	1.039	1.015	1.040	0.999	1.027
Chongqing	0.993	0.937	0.977	0.975	0.998	0.985	1.014	1.032	1.045	1.036	0.981	1.016	1.020
Sichuan	1.032	1.019	1.011	1.078	1.018	1.013	1.034	1.058	1.035	1.037	1.009	1.003	1.010
Guizhou	1.000	1.033	1.039	1.071	1.025	1.028	1.012	1.026	0.973	0.986	1.012	0.967	1.003
Yunnan	1.022	0.992	1.038	1.035	1.009	1.035	1.022	1.013	0.913	1.005	1.166	0.859	0.843
Shannxi	1.018	1.019	1.012	1.055	0.987	1.026	1.011	1.013	1.007	1.002	1.001	0.987	0.974
Gansu	0.980	1.000	1.022	1.035	1.019	1.001	1.015	1.019	0.987	1.013	1.012	1.009	1.006
Qinghai	1.040	0.957	0.943	1.112	0.967	1.036	0.993	1.026	1.040	1.012	1.031	1.013	1.016
Ningxia	1.026	1.022	0.935	1.109	0.944	1.014	0.998	1.017	1.020	1.015	1.020	1.021	1.057
Xinjiang	0.982	1.012	1.018	1.077	0.980	1.006	1.011	1.003	1.029	0.991	0.994	1.200	1.014
*National average*	1.017	1.013	1.010	1.019	1.017	1.001	1.002	1.009	1.002	0.997	1.006	1.002	1.004

**Table 3:** Decomposition on cross-region EPI_WE_ in China.

Regions	2003–2005			2006–2010			2011–2015		
	EPI_WE_	EPITC_WE_	EPIEC_WE_	EPI_WE_	EPITC_WE_	EPIEC_WE_	EPI_WE_	EPITC_WE_	EPIEC_WE_
Beijing	1.068	1.068	1.013	1.059	1.051	1.022	0.966	0.989	0.979
Tianjin	1.008	1.008	1.000	1.000	1.000	1.000	1.000	1.000	1.000
Hebei	1.023	1.030	0.994	1.018	1.018	0.999	0.997	1.002	0.995
Shanxi	1.023	1.032	0.991	0.981	1.007	0.974	0.989	1.001	0.988
Inner Mongolia	1.042	1.027	1.014	1.018	1.037	0.982	1.020	1.018	1.002
Liaoning	1.004	1.004	1.000	1.002	1.011	0.991	0.993	1.006	0.987
Jilin	1.014	1.021	0.993	0.993	1.021	0.973	1.010	1.005	1.004
Heilongjiang	1.014	1.014	1.000	1.000	1.000	1.000	1.002	1.002	1.000
Shanghai	1.026	1.026	1.000	1.010	1.010	1.000	1.003	1.003	1.000
Jiangsu	1.020	1.020	1.000	1.011	1.015	0.996	1.011	1.014	0.997
Zhejiang	1.029	1.045	0.985	1.035	1.035	1.000	0.999	0.997	1.002
Anhui	1.015	1.017	0.998	1.001	1.006	0.995	0.986	0.995	0.992
Fujian	1.023	1.035	0.989	1.012	1.014	0.998	1.008	1.009	0.999
Jiangxi	0.985	1.028	0.958	1.002	1.007	0.996	1.023	1.001	1.022
Shandong	1.007	1.007	1.000	1.001	1.001	1.000	1.000	1.000	1.000
Henan	1.018	1.013	1.004	0.973	1.005	0.968	0.994	1.002	0.992
Hubei	1.028	1.021	1.007	1.013	1.007	1.007	0.993	1.004	0.989
Hunan	1.001	1.001	1.000	0.993	1.002	0.991	0.984	1.002	0.982
Guangdong	1.016	1.016	1.000	1.001	1.001	1.000	0.969	1.001	0.968
Guangxi	1.017	0.977	1.045	0.927	0.995	0.932	1.022	1.001	1.020
Hainan	0.991	1.027	0.966	1.036	1.032	1.004	1.024	1.017	1.008
Chongqing	0.969	1.010	0.960	1.001	1.003	0.998	1.020	1.002	1.018
Sichuan	1.021	1.022	0.999	1.040	1.024	1.016	1.019	1.004	1.015
Guizhou	1.024	1.022	1.001	1.032	1.025	1.007	0.988	1.002	0.986
Yunnan	1.017	1.026	0.991	1.023	1.026	0.996	0.957	0.978	0.996
Shannxi	1.016	1.027	0.990	1.018	1.021	0.998	0.994	1.001	0.993
Gansu	1.001	1.034	0.968	1.018	1.029	0.989	1.005	1.002	1.003
Qinghai	0.980	1.027	0.954	1.027	1.027	1.000	1.023	1.013	1.010
Ningxia	0.994	1.017	0.978	1.016	1.016	1.001	1.026	1.008	1.018
Xinjiang	1.004	1.031	0.975	1.015	1.033	0.983	1.045	0.977	1.082

*Note*. Theoretically speaking, EPI_WE_ equals the product of EPITC_WE_ and EPIEC_WE_. However, given that all the figures in the table are multiyear mean values, EPI_WE_ does not equal to the product of EPITC_WE_ and EPIEC_WE_ sometimes. Fortunately, this situation is rare.

## Appendix

Results of EPI_WE_ for 30 administrative provinces from 2003 to 2015 are shown in Table 2.
